# Stress Distributions for Hybrid Composite Endodontic Post Designs with and without a Ferrule: FEA Study

**DOI:** 10.3390/polym12081836

**Published:** 2020-08-16

**Authors:** Pietro Ausiello, Antonio Gloria, Saverio Maietta, David C. Watts, Massimo Martorelli

**Affiliations:** 1 School of Dentistry—University of Naples Federico II, 80131 Naples, Italy; pietausi@unina.it; 2 Institute of Polymers, Composites and Biomaterials—National Research Council of Italy, 80125 Naples, Italy; 3 Department of Industrial Engineering, Fraunhofer JL IDEAS—University of Naples Federico II, 80125 Naples, Italy; smaietta@unina.it (S.M.); massimo.martorelli@unina.it (M.M.); 4 School of Medical Sciences and Photon Science Institute, University of Manchester, Manchester M13 9PL, UK; david.watts@manchester.ac.uk

**Keywords:** reverse engineering, Computer-Aided Design, finite element analysis, polyetherimide composites, endodontic post design

## Abstract

The aim of the current work was to analyze the influence of the ferrule effect for hybrid composite endodontic post designs consisting of carbon (C) and glass (G) fiber-reinforced polyetherimide (PEI), in upper canine teeth. Starting from theoretical designs of C-G/PEI hybrid composite posts with different Young’s moduli (Post **A**—57.7 GPa, Post **B**—31.6 GPa, Post **C**—graduated from 57.7 to 9.0 GPa in the coronal–apical direction) in endodontically treated anterior teeth, the influence of the ferrule effect was determined through finite element analysis (FEA). On the surface of the crown, a load of 50 N was applied at 45° to the longitudinal axis of the tooth. Maximum principal stresses were evaluated along the C-G/PEI post as well as at the interface between the surrounding tooth structure and the post. Maximum stress values were lower than those obtained for the corresponding models without a ferrule. The presence of a ferrule led to a marked decrease of stress and gradients especially for posts **A** and **B**. A less marked effect was globally found for Post **C**, except in a cervical margin section along a specific direction, where a significant decrease of the stress was probably due to local geometric features, compared to the model without a ferrule. The presence of a ferrule did not generally provide a marked benefit in the case of the graduated Post **C**, in comparison to other C-G/PEI posts. The outcomes suggest how such a hybrid composite post alone should be sufficient to optimize the stress distribution, dissipating stress from the coronal to the apical end.

## 1. Introduction

The restoration of root filled teeth plays an important role in clinical practice, and post placement has been widely investigated for strengthening teeth. The longevity of a tooth restored with a post-core system depends upon the post length and material, as well as on the applied load, post length and width of the root wall, and the presence—or not—of a ferrule and attachment to the root tissues [1,2]. In endodontically treated teeth, the generated levels of strain and stress are dependent upon the employed post-core systems [3].

Endodontists utilize many post-core systems for clinical use, with posts fabricated from several materials, in different sizes and shapes.

Dental posts are designed with either parallel or tapered forms and many are fabricated with anisotropic materials such as fiber-reinforced composites (FRCs) [4,5,6,7].

Many investigations considered materials that could reduce stresses in this context. However, neither stiff nor flexible posts represent the ideal solution.

Rigid (high modulus) metal posts generate high stress concentrations at the post–dentin interface [3,4] particularly in the cervical and apical regions of the tooth. However, flexible posts may cause stress concentrations within dentin [3,4].

The role of the geometry (i.e., length, diameter) and mechanical properties (i.e., stiffness) of the post was analyzed previously [8,9].

The positive influence of a ferrule on the strength of post-restored teeth and the contribution of the post material–shape combination were also demonstrated [10].

Efforts have been made to develop posts from functionally graded materials with tailored properties to optimize stress distribution, thus overcoming the drawbacks of either flexible or rigid posts [3,11].

A recent design of a hybrid composite post was created with tailored mechanical properties: Young’s modulus varying from 57.7 to 9.0 GPa in the coronal–apical direction.

This consisted of a thermoplastic polymer (polyetherimide—PEI) reinforced with carbon (C) and glass (G) fibers [12]. Finite element analysis (FEA) showed the benefits of tailoring the post mechanical properties within endodontically treated teeth [12].

The aim of the present research was to assess the influence of the ferrule effect with hybrid composite endodontic post designs for anterior teeth.

Specifically, an analysis on the effect of a ferrule on the stress distribution along the post and at the interface between the post and the surrounding structure was to be performed in teeth restored with C-G/PEI posts with different Young’s moduli.

The results could be critically compared to those obtained previously for designs without a ferrule [12].

## 2. Materials and Methods

Theoretical designs of hybrid composite posts consisting of a polyetherimide (PEI) matrix reinforced with carbon (C) and glass (G) fibers were previously reported and identified as follows [12]: Post **A** (C-G/PEI with a Young’s modulus of 57.7 GPa), Post **B** (C-G/PEI with a Young’s modulus of 31.6 GPa), Post **C** (C-G/PEI with a Young’s Modulus varying from 57.7 to 9.0 GPa in the coronal–apical direction). The following geometrical details were to be analyzed: 15-mm-long hybrid composite posts with a conical-tapered shape (length on coronal part—7 mm; length on conicity part—8 mm; coronal diameters—Ø 1.05, Ø 1.25, and Ø 1.45; and apical diameters—Ø 0.55, Ø 0.75, and Ø 0.95). The theoretical post design was created with the aid of finite element analysis (FEA), considering varied ply drop-off and stacking sequences, as well as using insights from the development of a C-G/PEI composite stem for a hip prosthesis with tailored properties along the head-tip direction [13,14]. Hand lay-up techniques, compression molding and water jet technology were employed to fabricate the C-G/PEI stem for a hip prosthesis [13,14].

In brief, the hybrid composite device consisted of four meaningful block-zones (I, II, III, IV) as already described [13,14]. The position, the orientation, and the distance of the carbon and glass fiber-reinforced plies (fiber volume fraction of 60%) from the middle plane were properly defined [13,14]. The distance of the carbon-reinforced plies from the middle plane decreased in the head-tip direction (i.e., from zone I to III) [13,14]. Even though zones I, II, and III consisted of both the carbon and glass fiber-reinforced plies, zone IV (i.e., tip) was only glass fiber-reinforced PEI.

Thus, Post **A** and Post **B** corresponded to zone I and zone II, respectively, of the previous developed hybrid composite hip stem, whereas Post **C** was designed as a C-G/PEI post with a Young’s Modulus decreasing from 57.7 to 9.0 GPa in the coronal–apical direction, reproducing the functionally graded structural design of the composite hip stem (i.e., from zone I to IV) [12,13,14].

Starting from an intact tooth (upper canine) and prior geometric models of endodontically treated anterior teeth [10,12], models with a 2.5 mm long and 0.5 mm wide ferrule (Young’s modulus and Poisson’s ratio of 70 GPa and 0.30, respectively) [10] were incorporated. A cement layer of 0.1 mm thickness was also incorporated between the prepared crown and the abutment. This cement thickness was also added between the post and the canal wall. The periodontal ligament was modeled as a 0.25-mm thin layer around the root [10,12]. All the models were imported into HyperMesh^®^ (HyperWorks^®^—14.0, Altair Engineering Inc., Troy, MI, USA).

FEA was performed on the three models, each with a ferrule: Model **A** (tooth with Post **A**), Model **B** (tooth with Post **B**), Model **C** (tooth with Post **C**). The model components and their Young’s moduli and Poisson’s ratios are given in Table 1.

**Figure 1:**
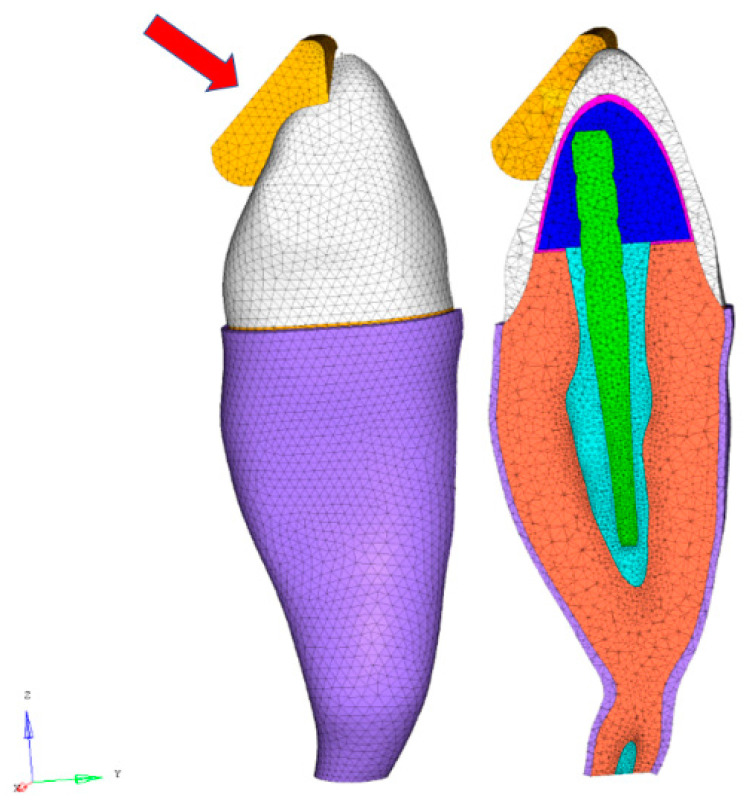
Finite element analysis (FEA) model according to the different components of the geometric models, mechanical properties and technical features.

As in the FEA of conceptual hybrid composite endodontic post designs in anterior teeth [12], a 3D mesh was generated and 3D solid CTETRA elements with four grid points were chosen. Adequate mesh size and mesh refinement techniques were used [10,12]. The total number of structural grids (56,026), elements excluding contact (234,011), node-to-surface contact elements (14,997), and degrees of freedom (223,578) were assigned for the three posts with a ferrule [10]. The closing phase of the chewing cycle with solid food (apple pulp) acting on the crown surface (Figure 1) was analyzed [10,12].

Furthermore, whereas slide-type contact elements were selected between the food and the tooth surface, “freeze” type elements were used as the contact condition between different parts of the post restoration [10,12]. To study the ferrule effect for hybrid composite posts, the post-restored teeth were analyzed under the same loading conditions as for models without a ferrule [12]. On the crown surface, a load of 50 N was applied at 45° to the longitudinal axis of the tooth (Figure 1). 

Linear elasticity was assumed, and a linear static analysis was carried out with a non-failure condition. Maximum principal stresses were analyzed along the post and at the interface between the post and the surrounding structure. The results were then compared with those previously obtained using the three C-G/PEI posts without a ferrule [12]. In the FEA results the color scale was chosen to allow for comparison between the models.

## 3. Results and Discussion

**Figure 2:**
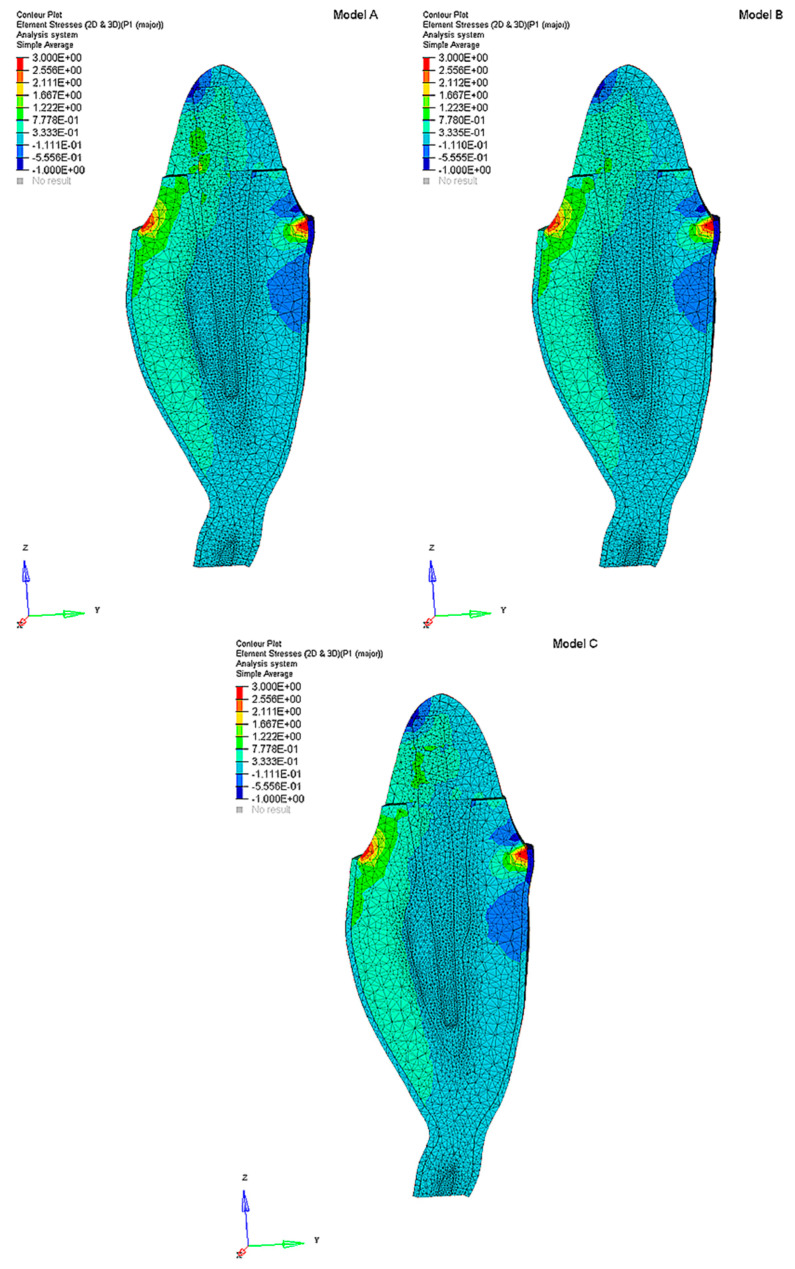
Models **A**, **B,** and **C**: maximum principal stress distribution (MPa) in the tooth restored with the three C-G/PEI posts with a ferrule: Model **A** (tooth with Post **A**—Young’s modulus of 57.7 GPa), Model **B** (tooth with Post **B**—Young’s modulus of 31.6 GPa), Model **C** (tooth with Post **C**—Young’s modulus decreasing from 57.7 to 9.0 GPa in the coronal–apical direction). The model components and their Young’s moduli and Poisson’s ratios are reported in Table 1.

The maximum principal stress distributions were analyzed in the abutment, post, post cement, root, and periodontal ligament. Cross sections were considered along the buccolingual direction for the three analyzed models (**A**, **B,** and **C**) (Figure 2).

**Figure 3:**
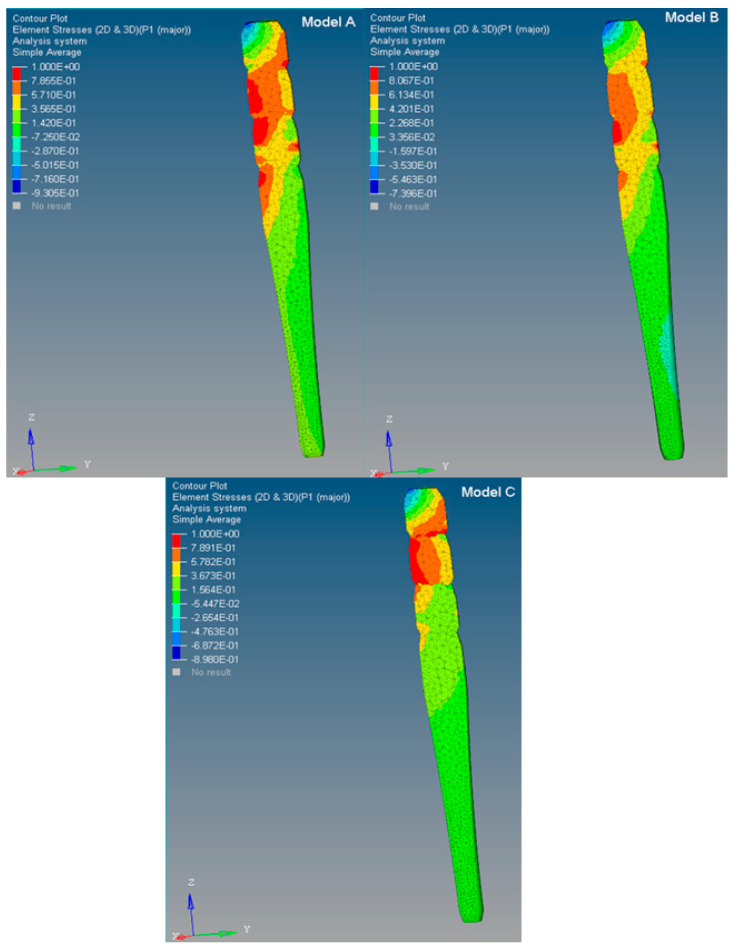
Maximum principal stress distribution (MPa) along the post in the restored tooth models (**A**, **B,** and **C**): mid-plane section.

**Figure 4:**
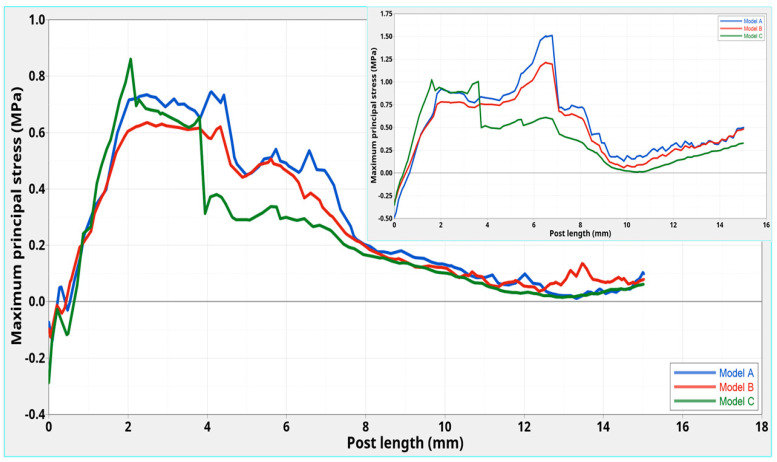
Models **A**, **B,** and **C**: maximum principal stress distributions along the center of the post from the coronal to the apical part. The inset is for corresponding models without a ferrule [12].

The stress distributions along the post were studied in the models of the tooth restored with the three C-G/PEI posts with a ferrule. (Figure 3 and Figure 4).

**Figure 5:**
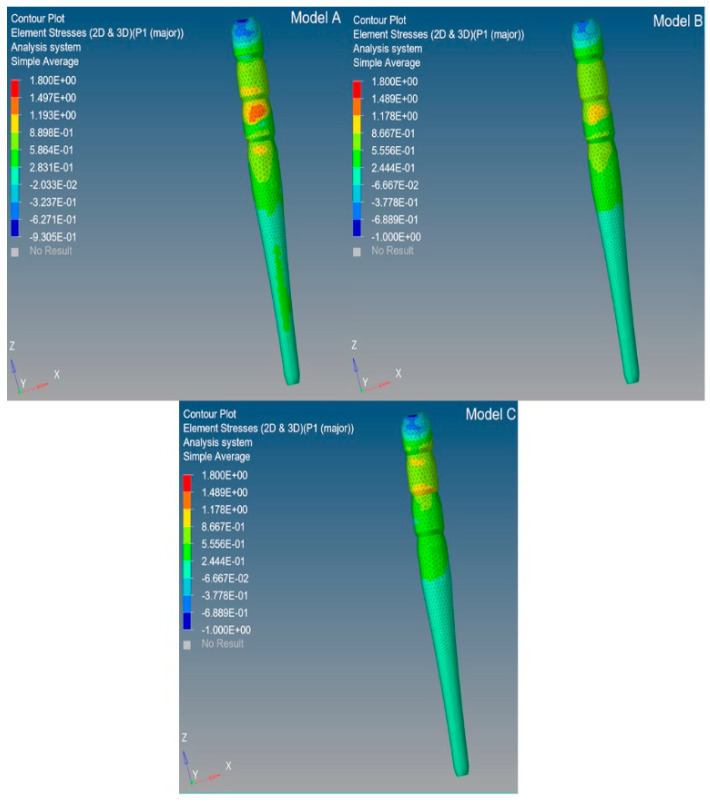
Models **A, B,** and **C**: maximum principal stress distribution at the interface between the post and surrounding structures from the coronal to the apical part.

**Figure 6:**
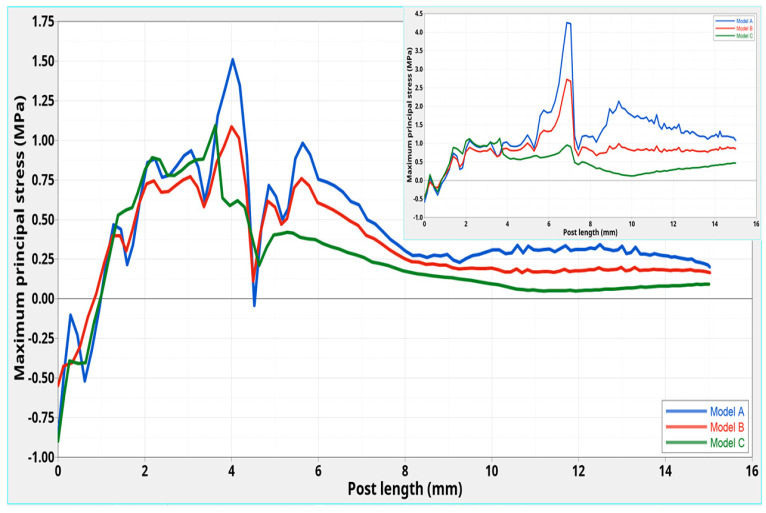
Models **A, B,** and **C**: maximum principal stress distributions at the interface between the post and surrounding structures from the coronal to the apical part. The inset is for corresponding models without a ferrule [12].

Different maximum principal stress distributions were evident along the three C-G/PEI posts. Even though, a higher stress (0.90 MPa) was initially found for Model **C** at 2.0 mm in the coronal part of the post, in comparison to models **A** (0.72 MPa) and **B** (0.60 MPa), consistently lower stresses were observed from 4.0 to 8.0 mm along the post in Model **C**. No great differences were then found from 8.0 mm to the apical part. However, at the interface between post and tooth, higher stress gradients were apparent especially for Model **A** in comparison to **B** and **C** (Figure 5 and Figure 6).

For all the models, starting from the coronal area, the maximum principal stress increased towards the cervical margin. However, the distributions (Figure 6) were different from those with C-G PEI posts without a ferrule (Figure 6—inset).

Higher stresses occurred for Model **A** at 3.5–4.0 mm, from the coronal end (Figure 6). A maximum of 1.15 MPa was found for models **C** and **B** at 3.5 and 4.0 mm, respectively, which was lower than for Model **A** (1.50 MPa at 4.0 mm).

In models without a ferrule [12], a maximum stress of 1.00 MPa was obtained for **C**, whereas higher values were found for models **A** (4.25 MPa) and **B** (2.75 MPa) shifted to about 7.0 mm.

After a small transition region, from 4.5 mm to the apical part, the stress for Model **C** was lower than for **A** and **B**. In this region, changes and fluctuations up to the apical end were less marked for **C** (Figure 6).

Thus, the presence of a ferrule shifted the maximum stress towards the coronal region and decreased stress. This was more marked for **A** and **B** than for **C**.

**Figure 7:**
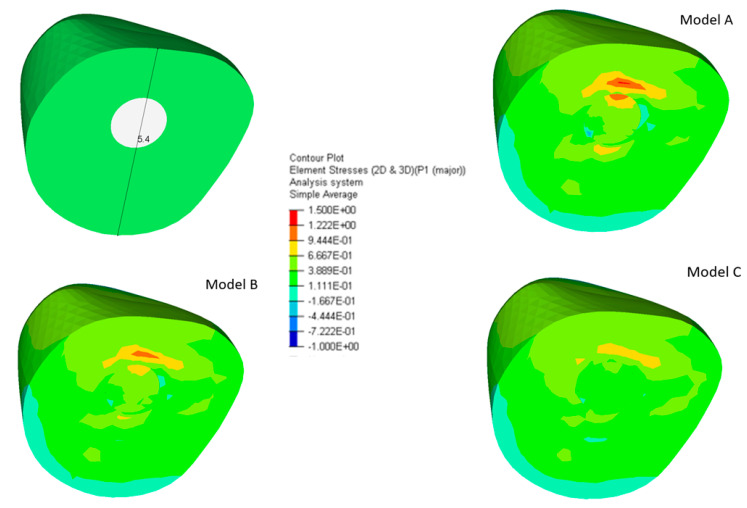
Cross sections at the cervical margin of the tooth (upper left) and maximum principal stress distributions for the three models.

The maximum principal stress for a cross section at the tooth cervical margin is shown in Figure 7.

**Figure 8:**
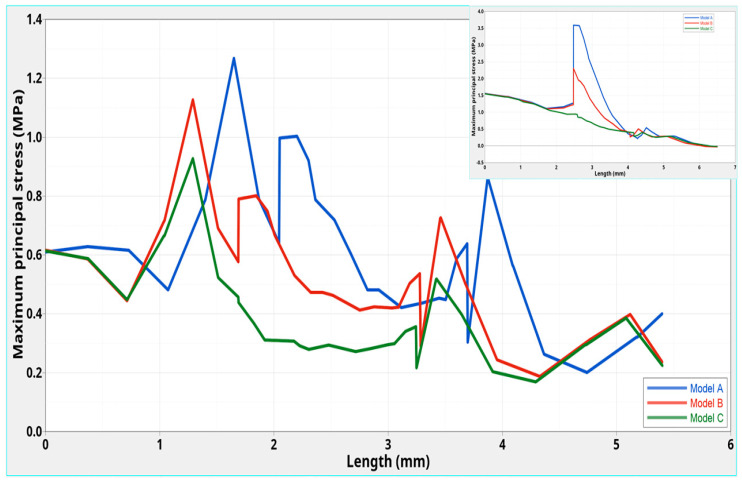
Maximum principal stress distributions at the interface between the post and tooth: analysis performed at the cervical margin of the tooth and evaluation of stress distribution along the direction indicated by the black line in Figure 7 (upper left). The inset reports the results obtained for corresponding models without a ferrule [12].

Figure 8 reports the stress distribution in the tooth cervical margin section, along the direction indicated by the black line in the schematic representation (Figure 7—upper left).

From 0 to 5.4 mm along the considered direction (Figure 7—upper left), maximum stresses of about 0.90 MPa and 1.10 MPa were found at 1.3 mm for models **C** and **B**, respectively, at the interface between post and tooth (Figure 8).

Model **A** exhibited a maximum of about 1.30 MPa, which was shifted to 1.7 mm along the considered direction, as compared to models **B** and **C**. As expected, high stress gradients were evident for models **A** and **B**. The obtained maxima were lower than those for the corresponding models without a ferrule (3.60, 2.30 and 1.60 MPa for models **A**, **B,** and **C,** respectively) [12] (Figure 8—inset).

Reverse engineering, theoretical, and experimental methodologies have been widely employed for the analysis of the stress distributions and mechanical behavior of restored teeth [9,10,11,12,15,16,17,18].

The use of a dental post fabricated using a high modulus material negatively influences the biomechanical behavior of a restored tooth, also causing vertical root fractures [1,3,11].

Ideally, a post should allow for the core stabilization without weakening the root [3,11]. An appropriate material-shape combination should focus on the stress transfer mechanism avoiding high stress concentrations [2], which are directly related to the stiffness mismatch between a post and surrounding structures [19,20,21].

The potential for using composite materials has been widely reported in different application fields [22,23,24]. In the case of hybrid polymeric systems, the control of crystallinity has a great impact on their properties and is fundamental for developing innovative functional materials [23]. Moreover, the effects of volume fraction, size, and shape of the reinforcement on the composite properties have been largely stressed [24].

Several post-core systems (i.e., prefabricated fiberglass posts with resin cores) are currently employed, the aim being to prevent root fracture [4]. Furthermore, composite posts with different shapes have been designed using carbon (C), glass (G), and quartz fibers.

Clinical procedures have been continuously modified to restore endodontically-treated teeth [25,26] and scientific studies on the performance of these systems are increasing. The selection of the fiber post and the proper preparation of the root canal represent the first steps in the clinical application.

From a clinical point of view, a post is generally selected taking into account the remaining tooth structure and the functional demands. Both in the case of minimal radicular tooth structure and pulpless teeth, glass or carbon fiber posts are usually deployed [27]. Interesting clinical results and no root fractures were obtained with teeth restored using these posts [27,28,29].

Although many in vitro and clinical studies have been carried out in this field, there are still no precise recommendations [1,20,26,27]. It is frequently reported that endodontically treated teeth may present a higher risk of biomechanical failure if compared to teeth, and the price for using posts may be an increased risk of damaged tooth structure [1,3,11,27,28,29,30]. Three-dimensional sealing of the root canal space also represents one of the main challenges of the endodontic therapy and it is a crucial requisite to prevent apical and coronal microleakage. For this reason, the effect of post-space preparation on the sealing ability has been suitably studied with a special focus on the bacterial leakage [31].

However, in the field of total hip arthroplasty some studies [13,14] have investigated hybrid composite materials consisting of carbon and glass fiber-reinforced polyetherimide (PEI). Such C-G/PEI hybrid composites have been manufactured with different shapes and functionally graded stiffness along the axial length [14]. Even if the fabrication of large stems for hip prostheses in a hybrid design is relatively straightforward, new emerging microfabrication technologies should make it possible to manufacture endodontic posts with analogous hybrid PEI composite designs [12]. Standard C-G/PEI composite specimens, which corresponded to the four meaningful block-zones, were fabricated and experimentally tested (e.g., tensile, flexural and torsional tests). As an example, the bending modulus varied from 57.70 ± 0.31 GPa (zone I) to 31.60 ± 1.18 GPa (zone II), 10.90 ± 0.97 GPa (zone III) and 9.05 ± 0.54 GPa (zone IV), whereas the yield stress ranged from 1051.0 ± 70.1 MPa (zone I) to 943.0 ± 52.3 MPa (zone II), 473.0 ± 25.8 MPa (zone III), and 389.0 ± 21.1 MPa (zone IV) [14]. The mechanical properties evaluated for the standard specimens clearly reflect a principle of material design, which is a peculiar feature of the composite systems.

Similarly, much research has been devoted to the development of fiber-reinforced posts, as well as to the conceptual design of inhomogeneous dental posts [3] and hybrid composite posts (C-G/PEI) where the Young’s modulus varied in the coronal–apical direction [12].

The theoretical designs involved equations assigning the Young’s moduli and Poisson’s ratios as a function of distance along the neutral axis of the device [3], as well as an advanced design modulating the distance of carbon and glass fiber-reinforced plies from the middle plane in the coronal–apical direction [12].

When the ferrule was not incorporated in the models, previous work [12] evidenced a mostly uniform stress distribution with no significant stress concentration in the teeth restored using the C-G/PEI post with a modulus varying from 57.7 to 9.0 GPa in the coronal–apical direction (Post **C**).

Differently from a previous work concerning the effect of a ferrule on the mechanical behavior of canine teeth restored with a quartz fiber post (conical–tapered shape) and a carbon fiber post (conical–cylindrical shape) [10], the current analysis was focused on the use of C-G/PEI posts (conical–tapered shape) and evidenced how the presence of ferrule may alter the maximum principal stress distribution, compared to results for the models without a ferrule [12].

The analysis demonstrated that also in the case of posts with a ferrule, Model **C** continued to show lower stresses with no significant stress concentrations, compared to models **A** and **B**.

In addition, with regard to Model **C**, the presence of ferrule provided a different stress distribution along the center of the post and stresses (0.90–0.20 MPa) that were slightly lower than those found in the corresponding model without a ferrule (1.00–0.31 MPa) [12] (Figure 4—inset). A similar observation may be made for the stress distribution at the interface between the Post **C** and surrounding structures.

However, looking at the maximum principal stress distributions along the post (Figure 4) and at the interface (Figure 6) for models **A** and **B**, the presence of ferrule decreased the stress for each model when compared to no ferrule [12]. This decrease was much more marked for models **A** and **B** than that found for Model **C**.

Thus, in the case of C-G PEI posts with a constant Young’s modulus (Post **A**—57.7 GPa, Post **B**—31.6 GPa) the ferrule effect led to a significant decrease of stress and gradients.

By contrast, the presence of a ferrule did not markedly alter the previously known role of the functionally graded structural design of Post **C** in reducing stress and minimizing stress concentrations [12].

Consequently, an appropriate material–design combination, involving the tailored distances of carbon and glass fiber-reinforced plies from the middle plane in the coronal–apical direction, as well as the presence of only glass fiber-reinforced plies at the apical end of the post [12], alone should be already able to improve (reduce) stress distributions.

Such results were partially confirmed by further analyses of the maximum principal stress distribution, which were performed in a cross section at the cervical margin of the tooth along a specific direction (Figure 7 and Figure 8), where the ferrule effect would seem to play a more significant role as lower stresses were also attained for Model **C** in comparison to those found for the corresponding model without ferrule [12]. In this region, the presence of a ferrule clearly affected the geometry of the system and, unlike the stress distribution along the center of post and at the interface with the surrounding structures, the local geometric features would seem to provide an important contribution, leading to a significant decrease of the stress also for Model **C**, compared to the corresponding model without a ferrule [12].

In the present study FEA was employed to provide an analysis of the influence of the ferrule effect for C-G/PEI posts in endodontically treated anterior teeth. However, this does not properly take into account all the factors present in the oral environment (i.e., saliva, water, blood), which affect the post-dentin bonding [4]. For this reason, the current investigation should be verified and supplemented by clinical studies.

Further potential limitations of the present study were: (i) the assumption of a constant Young’s modulus for the periodontal ligament; and (ii) the linear static analysis performed with a non-failure condition.

The use of posts in the restoration of root filled teeth is both controversial and challenging because of the confusing range of opinions reported by researchers and dentists. This makes it complex to choose the most appropriate restorative approach, in terms of materials, devices, and clinical procedures [1,2,26,27,30].

Clinical findings have been analyzed about post usage in “ferruled” and “unferruled” teeth to better understand the separate contributions of post and ferrule [30]. A positive effect of the post was not confirmed in many cases, whereas the ferrule effect and the possibility of maintaining the cavity walls were often indicated as the main contributing factors to the restoration and tooth survival in endodontically treated teeth [30]. Although much emphasis has been given to the ferrule effect and endodontic post usage, today unequivocal and clear guidelines are missing [30].

## 4. Conclusions

Within the limitations of the current research, some conclusions were reached. A marked decrease of stress and gradients was found for C-G/PEI posts with a constant Young’s modulus, compared to the same models without ferrule. Furthermore, the presence of ferrule did not markedly improve (reduce) stress distributions when using the C-G/PEI post with a graduated modulus varying from 57.7 to 9.0 GPa in the coronal–apical direction. The post alone was already sufficient to dissipate stress from the coronal to the apical end.

However, strong limitations are clearly related to FEA which can make an overall conclusion about the selected C-G/PEI post extremely difficult as experimental tests must be performed and compared to the results obtained from simulations.

The obtained findings should probably help to improve predictions of the impact of hybrid composite posts with tailored properties in dentistry research as well as in clinical practice.

Anyway, this research can contribute to provide a further insight into the design of dental posts, even if some concerns remain, especially about the clinical recommendations, the potential fractures in endodontically treated teeth restored with posts, and the impact of post space preparation on coronal microleakage.

## Figures and Tables

**Table 1:** Young’s moduli and Poisson’s ratios for different model components.

Component	Young’s Modulus (GPa)	Poisson’s Ratio
Lithium disilicate crown	70	0.30
Crown cement	8.2	0.30
Abutment	12	0.30
Post A	57.7	0.30
Post B	31.6	0.30
Post C	57.7–9.0 *	0.30
Post cement	8.2	0.30
Root	18.6	0.31
Periodontal ligament	0.15 (× 10^−3^)	0.45
Food (apple pulp)	3.41 (× 10^−3^)	0.10

* For post **C**, the modulus decreased in the coronal–apical direction [12].
